# Trends and disparities in peptic ulcer disease-related mortality in the United States from 1999 to 2020: A cross-sectional study

**DOI:** 10.1097/MD.0000000000042129

**Published:** 2025-10-03

**Authors:** Muhammad Babar, Mohsin Raza, Muhammad Asfandyar Nadir, Ariba Fida, Izzah Fayyaz, Eeman Ahmad, Armaghan Ur Rehman, Zain Ali Nadeem, Muhammad Bilal Sardar, Arsalan Nadeem, Akash Gupta, Raheel Ahmed

**Affiliations:** a Department of Surgery, Services Hospital Lahore, Lahore, Pakistan; b Department of Medicine, Allama Iqbal Medical College, Lahore, Pakistan; c Department of Medicine, Fatima Memorial Hospital College of Medicine and Dentistry, Lahore, Pakistan; d Department of Emergency Medicine, Trinity Health Livonia; e Department of Cardiac Function, National Heart and Lung Institute, Imperial College London, London, UK.

**Keywords:** deaths, mortality, peptic ulcer disease

## Abstract

Peptic ulcer disease (PUD) is associated with severe complications such as hemorrhage and perforation, leading to high morbidity and mortality rates despite advancements in treatment. This study aims to evaluate the temporal and regional mortality trends of adults with PUD in the United States from 1999 to 2020, stratified by sex, race/ethnicity, age group, and geographic region. Utilizing death certificate data from the Centers for Disease Control and Prevention Wide-ranging Online Data for Epidemiologic Research database, age-adjusted mortality rates (AAMRs) per 100,000 individuals were derived from crude mortality rates (CMRs). Trends in AAMRs were analyzed through annual percent change (APC) and average APC (AAPC) using Joinpoint regression (Joinpoint Regression Program, V5.0.2). From 1999 to 2020, a total of 37,471 deaths due to PUD were reported in the United States. The AAMR remained stable from 1999 to 2001 (APC: 0.59), followed by a sharp decline from 2001 to 2012 (APC: −11.79). After a period of stability from 2012 to 2018 (APC: 1.53), the trend reversed with an increase from 2018 to 2020 (APC: 8.45). Males had higher AAMR (0.84) than females (0.71). Non-Hispanic Whites had the highest AAMR (0.82), and Hispanics or Latinos the lowest (0.49), with an overall decrease in mortality rates across all racial groups, particularly among Non-Hispanic Black or African Americans. The West had the highest AAMR (0.87), and the Northeast the lowest (0.71), with all regions showing a downward trend. Rural areas exhibited a higher AAMR (0.86) compared to urban areas (0.76). Mortality was most concentrated among adults aged 85 years and older (CMR: 7.63), and the lowest CMR was recorded in the 25 to 54 years age group (0.16). Most deaths occurred in medical facilities (81.89%), followed by decedents’ homes (8.36%) and nursing homes/long-term care (3.85%). The District of Columbia (AAMR: 1.47) reported the highest AAMR, while New Jersey (0.59) had the lowest. Despite the overall decline, regional and demographic disparities remain, highlighting the need for continued efforts to address PUD-related mortality.

## 1. Introduction

Peptic ulcer disease (PUD) remains a significant global health concern, affecting around 99.4 per 100 000 people worldwide in 2019.^[1]^ PUD can lead to severe complications such as hemorrhage or perforation, which may contribute to substantial morbidity and mortality rates. Research indicates that bleeding peptic ulcers, a prevalent cause of emergency hospital admissions, can result in mortality rates as high as 10%.^[2]^ Similarly, perforated peptic ulcers present dire risks, especially in emergency scenarios, where factors such as shock and postoperative complications critically influence patient outcomes.^[3]^ Despite advancements in therapeutic interventions, the mortality associated with PUD complications continues to be a formidable challenge, particularly in cases of severe bleeding.^[4]^

The management of PUD, especially in case of perforation, is crucial due to the associated high mortality rates.^[5]^ Various determinants including age, comorbid conditions, and the underlying cause—such as smoking, H. pylori infection, and non-steroidal anti-inflammatory drugs (NSAIDs)–significantly impact mortality risk in PUD patients.^[4]^ In response to these challenges, scoring systems such as the Predictive Score of Mortality in Perforated Peptic Ulcer and the Boey score have been developed to enhance the accuracy of mortality predictions in patients suffering from peptic ulcer perforation.^[6]^

While the global prevalence of PUD seems to be declining over the years,^[1]^ contemporary data on PUD-related mortality trends in the United States (US) is not available. This paper aims to address the critical issue of PUD-related mortality trends in US from 2001 to 2020 stratified by sex, age, race or ethnicity, census region, urbanization, state, and place of death. This would help modify current management strategies to better suit the high-risk groups and aid in the development of improved interventions and strategies that can significantly reduce the mortality rates associated with PUD.

## 2. Methods

This study conforms to the Strengthening the Reporting of Observational Studies in Epidemiology (STROBE) statement.^[7]^

### 2.1. Study setting and population

We queried the Centers for Disease Control and Prevention Wide-Ranging Online Data for Epidemiologic Research (CDC WONDER) database for records of all death certificates with PUD as a contributing cause of death or the underlying cause of death in from 1999 to 2020 using the International Classification of Diseases (ICD)-10 codes: K25.1, K25.2, K25.5, K25.6, K26.1, K26.2, K26.5, K26.6.^[8]^ Only adults 25 years or older were included, as this range is commonly used to represent adult populations in database studies.^[9]^ We used the Multiple Cause of Death database to ensure all cases of PUD-related deaths were captured.

### 2.2. Study design

Data were collected for mortality stratified by sex, race or ethnicity, age groups, census regions, states, urbanization, and place of death. Sex was considered male or female as written on the death certificate. Race/ethnicity was classified into non-Hispanic (NH) White, NH Black or African American, Hispanic or Latino, NH American Indian or Alaskan Native, and NH Asian or Pacific Islander, as reported on the death certificates. The individuals were divided by age into 3 groups: 25 to 54 years, 55 to 84 years, and 85 + years. The regions included Northeast, Midwest, South, and West according to the U.S. Census Bureau definitions, and the National Center for Health Statistics Urban-Rural Classification Scheme was used to describe urban and rural areas per the 2013 US census classification.^[10]^ Institutional review board approval was not required because the study utilized deidentified public use data.

### 2.3. Statistical analysis

To examine the temporal trends for PUD-related mortality, we calculated crude mortality rates (CMRs) and age-adjusted mortality rates (AAMRs) per 100,000 population with 95% confidence intervals (CIs). CMRs were calculated by dividing the number of deaths in a year by the corresponding US population of that year. We then standardized the deaths to the US population in the year 2000 to obtain AAMRs.^[11]^ We analyzed the temporal trends using the Joinpoint Regression Program (Joinpoint V 5.1.0.0, National Cancer Institute) to calculate the annual percent change (APC) and the average annual percentage change (AAPC) with 95% CIs in AAMR or CMR (12). This method fits log-linear regression models to identify significant changes in AAMR, creating trend segments connected by joinpoints, with each joinpoint representing years where significant variation occurred. The advantage of AAPCs calculated by Joinpoint regression is that they account for trend transitions in contrast to conventional APC [https://pmc.ncbi.nlm.nih.gov/articles/PMC2843083/#sec8]. Joinpoint regression has been previously used in several epidemiological studies to describe mortality trends over time [https://pubmed.ncbi.nlm.nih.gov/30862942/, https://pubmed.ncbi.nlm.nih.gov/36551741/, https://pubmed.ncbi.nlm.nih.gov/39854486/].

The APCs we considered increasing or decreasing if the slope for the change in mortality significantly differed from zero using *t*-test. CMRs were used to analyze the age groups, as AAMRs could not be calculated.

## 3. Results

**Figure 1.**
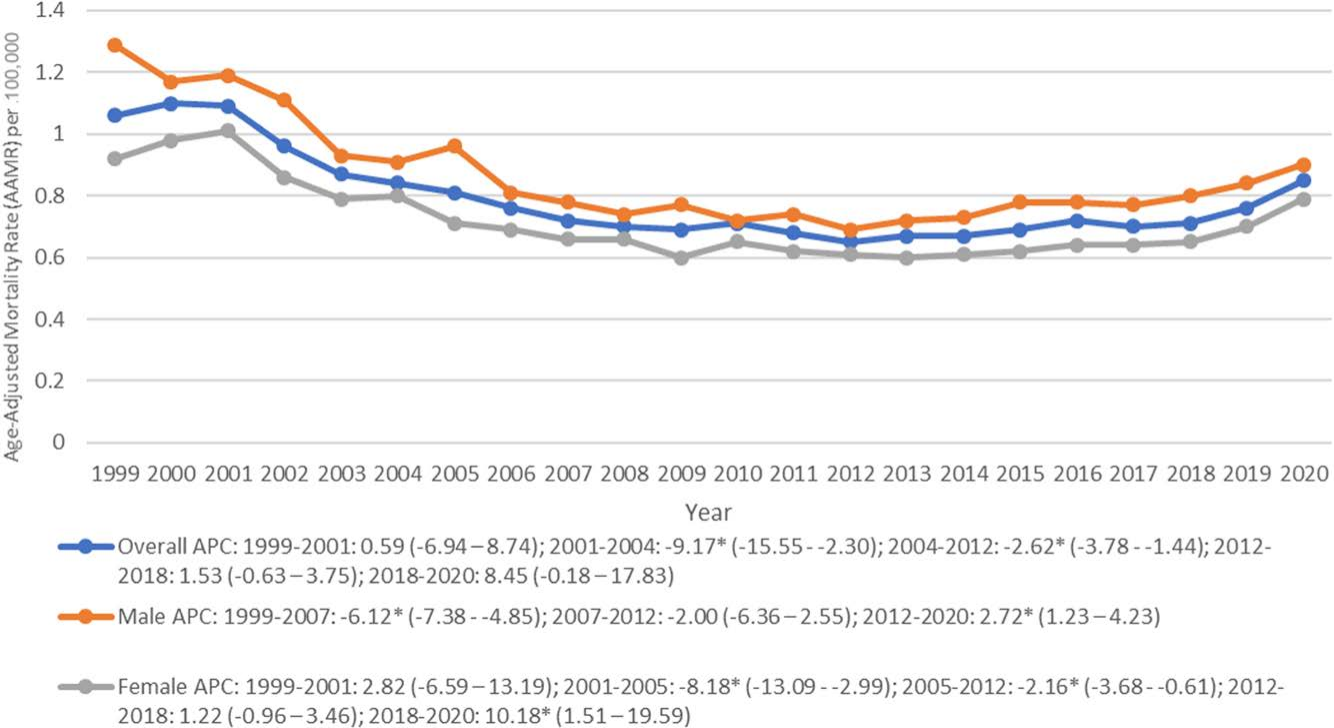
Overall and sex-stratified peptic ulcer disease-related age-adjusted mortality rates per 100,000 among adults in the United States, 1999 to 2020 (APC = annual percentage change).

A total of 37,471 deaths were reported in adults with PUD in the US (AAMR: 0.76, 95% CI: 0.75 to 0.77) from 1999 to 2020 (Table 1). The AAMR remained stable from 1999 to 2001 followed by a steep decrease from 2001 to 2012. Thereafter, the trajectory remained stable from 2012 to 2020 (Fig. 1, Table 2; Table S1, Supplemental Digital Content, https://links.lww.com/MD/P16).

**Table 1:** Demographic characteristics of peptic ulcer disease-related among adults in the United States from 1999 to 2020.

Variable	Deaths (%)	Population	AAMR* (per 100,000)
Entire cohort	–	4473,854,489	0.76 (0.75–0.77)
Gender			
Female	53.00	2319,297,578	0.71 (0.70–0.72)
Male	47.00	2154,556,911	0.84 (0.83–0.85)
Census region			
Northeast	17.56	827,193,779	0.71 (0.69–0.73)
Midwest	23.39	969,567,311	0.80 (0.79–0.82)
South	35.07	1652,256,217	0.76 (0.74–0.77)
West	23.98	1024,837,182	0.87 (0.85–0.89)
Race/ethnicity			
NH† American Indian or Alaska Native	0.55	33,081,153	0.71 (0.61–0.81)
NH Asian or Pacific Islander	2.61	237,142,712	0.51 (0.48–0.55)
NH Black or African American	9.87	518,937,524	0.75 (0.73–0.78)
NH White	86.98	3095,342,890	0.82 (0.81–0.83)
Hispanic or Latino	–	589,350,210	0.49 (0.47–0.52)
Urbanization			
Urban	–	3795,213,822	0.76 (0.75–0.76)
Rural	–	678,634,169	0.86 (0.84–0.88)
Age groups‡			
25 to 54 yr	–	2778,952,977	0.16 (0.15–0.16)
55 to 84 yr	–	1575,387,621	1.52 (1.50–1.54)
≥85 yr	–	119,513,891	7.63 (7.47–7.78)
Place of death			
Medical facility	81.9	–	–
Decedent’s home	8.36	–	–
Nursing home/ long-term care	3.85	–	–
Hospice facility	3.33	–	–
Other	2.28	–	–
Place of death unknown	0.29	–	–

* Age-adjusted mortality rate.

† Non-Hispanic.

‡ Crude Mortality Rate is used for analysis instead of age-adjusted mortality rates for age groups.

**Table 2:** Annual percentage changes (APCs) and average annual percentage changes (AAPCs) in peptic ulcer disease-related among adults in the United States from 1999 to 2020.

Variable	Trend segment	Lower endpoint	Upper endpoint	APC* (95% CI)	AAPC† (95% CI)	*P* value
Overall	1	1999	2001	0.5982 (−6.9424 to 8.7498)	−1.1155 (−2.5247 to 0.314)	.125551
	2	2001	2004	−9.1725‡ (−15.5574 to −2.3048)		
	3	2004	2012	−2.6223‡ (−3.7879 to −1.4426)		
	4	2012	2018	1.5355 (−0.6385 to 3.7571)		
	5	2018	2020	8.4517 (−0.1802 to 17.83)		
Sex						
Female	1	1999	2001	2.8272 (−6.5902 to 13.1941)	−0.8151 (−2.3134 to 0.7062)	.291963
	2	2001	2005	−8.1806‡ (−13.0901 to −2.9938)		
	3	2005	2012	−2.1644‡ (−3.6894 to −0.6153)		
	4	2012	2018	1.223 (−0.9686 to 3.46320		
	5	2018	2020	10.1853‡ (1.5125 to 19.5992)		
Male	1	1999	2007	−6.1271‡ (−7.3846 to −4.8525)	−1.8487‡ (−3.0275 to −0.6556)	.002471
	2	2007	2012	−2.0061 (−6.3676 to 2.5586)		
	3	2012	2020	2.7277‡ (1.2393 to 4.2379)		
US Census Region						
Northeast	1	1999	2008	−6.6943‡ (−8.5358 to −4.8157)	−2.4806‡ (−3.5527 to −1.3965)	.000008
	2	2008	2020	0.8041 (−0.6468 to 2.2762)		
Midwest	1	1999	2007	−6.5550‡ (−8.8561 to −4.1959)	−1.2328 (−3.5502 to 1.1403)	.30585
	2	2007	2018	0.2404 (−1.8444 to 2.3695)		
	3	2018	2020	13.6256 (−9.3667 to 42.4508)		
South	1	1999	2013	−3.6830‡ (−4.2981 to −3.064)	−1.0695‡ (−1.7336 to −0.4009)	.00175
	2	2013	2020	4.3722‡ (2.5537 to 6.2229)		
	–	–	–	–		
West	1	1999	2008	−6.1330‡ (−7.6935 to −4.5462)	−2.2298‡ (−3.0751 to −1.3771)	<.000001
	2	2008	2020	0.8038 (−0.2455 to 1.8641)		
	–	–	–	–		
Race/ethnicity						
NH American Indian or Alaska Native	1	–	–	–	–	–
	2	–	–	–		
	3	–	–	–		
NH Asian or Pacific Islander	1	1999	2016	−3.6868‡ (−5.3403 to −2.004)	−1.4665 (−3.8905 to 1.0187)	.245043
	2	2016	2020	8.5551 (−3.72 to 22.3951)		
	3	–	–	–		
NH Black or African American	1	1999	2010	−6.0738‡ (−7.7305 to −4.3874)	−2.2834‡ (−3.4866 to −1.0653)	.000258
	2	2010	2020	2.0629‡ (0.039 to 4.1277)		
	3	–	–	–		
NH White	1	1999	2008	−5.1206‡ (−6.175 to −4.0544)	−1.036 (−2.1532 to 0.094)	.07222
	2	2008	2018	0.5877 (−0.538 to 1.7261)		
	3	2018	2020	10.2846 (−0.8477 to 22.6667)		
Hispanic or Latino	1	1999	2015	−4.1240‡ (−5.2756 to −2.9585)	−1.4176 (−2.9005 to 0.0879)	.064844
	2	2015	2020	7.7670‡ (1.8397 to 14.0392)		
	–	–	–	–		
Urbanization						
Urban	1	1999	2008	−5.4061‡ (−6.3283 to −4.4749)	−1.7497‡ (−2.6464 to −0.8448)	.000161
	2	2008	2014	−0.8029 (−3.3697 to 1.8321)		
	3	2014	2020	3.0089‡ (1.1553 to 4.8964)		
Rural	1	1999	2007	−4.9630‡ (−7.0335 to −2.8463)	−0.3984 (−2.3362 to 1.5778)	.690468
	2	2007	2018	0.2131 (−1.4251 to 1.8786)		
	3	2018	2020	16.1822 (−3.6361 to 40.0765)		
Age§						
25 to 54 yr	1	1999	2016	−1.9710‡ (−2.4534 to −1.4862)	−0.0783 (−0.967 to 0.8184)	.863563
	2	2016	2020	8.3820‡ (3.5165 to 13.4762)		
	–	–	–	–		
55 to 84 yr	1	1999	2009	−6.1708‡ (−7.1139 to −5.2181)	−1.5746‡ (−2.7223 to −0.4133)	.008003
	2	2009	2018	1.1008 (−0.2901 to 2.511)		
	3	2018	2020	10.8002 (−0.5719 to 23.4729)		
≥85 yr	1	1999	2008	−6.3261‡ (−7.8442 to −4.7829)	−2.9010‡ (−3.7357 to −2.0591)	<.000001
	2	2008	2020	−0.2503 (−1.3111 to 0.8219)		
	–	–	–	–		

* Annual percentage change.

† Average annual percentage change.

‡ The AAPC is significantly different from zero at the alpha = 0.05 level.

§ Crude rate is used for all age dependent analysis.

### 3.1. Trends by sex

Males consistently showed a higher AAMR (0.84, 95% CI: 0.83 to 0.85) than females (AAMR: 0.71, 95% CI: 0.70 to 0.72) (Table 1). From 1999 to 2020, an overall decrease in AAMR was observed in males (AAPC: −1.84, 95% CI: −3.02 to −0.65), but not in females (AAPC: −0.81, 95% CI: −2.31 to 0.70) (Fig. 1, Table 2; Table S1, Supplemental Digital Content, https://links.lww.com/MD/P16). From 1999 to 2007, a decrease in AAMR was observed in males which was followed by stable AAMRs till 2012 and a gradual increase from 2012 to 2020 (Fig. 1, Table 2; Table S1, Supplemental Digital Content, https://links.lww.com/MD/P16). From 1999 to 2001, females exhibited a stable AAMR which was followed by an abrupt decline from 2001 to 2012, and a period of stability from 2012 to 2018. From 2018 to 2020, females depicted a sharp rise in AAMR (Fig. 1, Table 2; Table S1, Supplemental Digital Content, https://links.lww.com/MD/P16).

### 3.2. Trends by race and ethnicity

**Figure 2.**
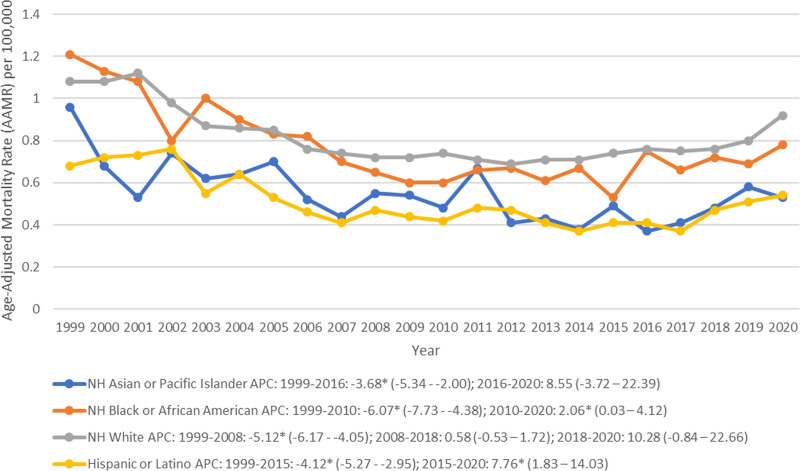
Peptic ulcer disease-related age-adjusted mortality rates stratified by race per 100,000 among adults in the United States, 1999 to 2020 (APC = annual percentage change, NH = non-Hispanic).

NH Whites exhibited the highest AAMR (0.82, 95% CI: 0.81 to 0.83) followed by NH Blacks or African Americans (0.75, 95% CI: 0.73 to 0.78), NH American Indians or Alaska Natives (0.71, 95% CI: 0.61 to 0.81), NH Asians or Pacific Islanders (0.51, 95% CI: 0.48 to 0.55) and finally Hispanics or Latinos who exhibited the lowest AAMR (0.49, 95% CI: 0.47 to 0.52) (Table 1). From 1999 to 2020, an overall decrease in the trends of AAMR was observed among all races with NH Blacks exhibiting the most significant decrease (AAPC: −2.28, 95% CI: −3.48 to −1.06) (Fig. 2, Table 2; Table S2, Supplemental Digital Content, https://links.lww.com/MD/P16). NH Whites exhibited a sharp decrease in AAMR from 1999 to 2008 (APC: −5.12, 95% CI: −6.17 to −4.05) after which the trajectory remained stable from 2008 to 2018 (APC: 0.58, 95%: 0.53 to 1.72) and from 2018 to 2020 (APC: 10.28, 95% CI: −0.84 to 22.66) (Fig. 2, Table 2; Table S2, Supplemental Digital Content, https://links.lww.com/MD/P16). NH Blacks depicted a sharp decline in trends (APC: −6.07, 95% CI: −7.73 to −4.38) from 1999 to 2010 which was followed by a significant rise (APC: 2.06, 95% CI: 0.03 to 4.12) between 2010 and 2020 (Fig. 2, Table 2; Table S2, Supplemental Digital Content, https://links.lww.com/MD/P16). Between 1999 and 2016, a decrease was observed in the trajectory for NH Asians (APC: −3.68, 95% CI: −5.34 to 2.00), followed by a period of stability from 2016 to 2020 (APC: 8.55, 95% CI: −3.72 to 22.39) (Fig. 2, Table 2; Table S2, Supplemental Digital Content, https://links.lww.com/MD/P16). Hispanics showed a general decrease in trends from 1999 to 2015 (APC: −4.12, 95% CI: −5.27 to −2.95) followed by a significant increase from 2015 to 2020 (APC: 7.76, 95% CI: 1.83 to 14.03) (Fig. 2, Table 2; Table S2, Supplemental Digital Content, https://links.lww.com/MD/P16).

### 3.3. Trends by census region and urbanization

**Figure 3.**
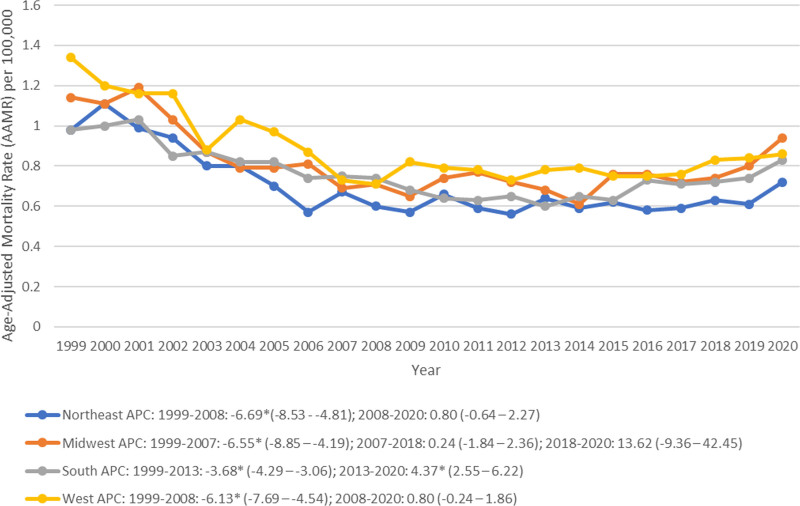
Peptic ulcer disease-related age-adjusted mortality rates stratified by census region per 100,000 among adults in the United States, 1999 to 2020 (APC = annual percentage change).

The West exhibited the highest AAMR (0.87, 95% CI: 0.85 to 0.89), followed by the Midwest (0.80, 95% CI: 0.79 to 0.82), the South (0.76, 95% CI: 0.74 to 0.77) and finally the Northeast (0.71, 95% CI: 0.69 to 0.73) (Table 1). Between 1999 and 2020, the Northeast showed the greatest decline (AAPC: −2.48, 95% CI: −3.55 to −1.39), followed by the West (AAPC: −2.22, 95% CI: −3.07 to −1.37) and the South (AAPC: −1.06, 95% CI: −1.73 to −0.40); stable mortality rates were observed in the Midwest (AAPC: −1.23, 95% CI: −3.55 to 1.14) (Fig. 3, Table 2; Table S3, Supplemental Digital Content, https://links.lww.com/MD/P16). The West showed a decrease in trends from 1999 to 2008 (APC: −6.13, 95% CI: −7.69 to −4.54) before achieving a stable trajectory from 2008 to 2020 (AAPC: 0.80, 95% CI: −0.24 to 1.86) (Fig. 3, Table 2; Table S3, Supplemental Digital Content, https://links.lww.com/MD/P16). The Midwest depicted a significant decrease in AAMR from 1999 to 2007 (APC: −6.55, 95% CI: −8.85 to −4.19), followed by stabilization of the trend from 2007 to 2018 (APC: 0.24, 95% CI: −1.84 to 2.36) and from 2018 to 2020 (APC: 13.62, 95% CI: −9.36 to 42.45) (Fig. 3, Table 2; Table S3, Supplemental Digital Content, https://links.lww.com/MD/P16). From 1999 to 2013, the South demonstrated a decrease in trends (APC: −3.68, 95% CI: −4.29 to −3.06) followed by an increase (APC: 4.37, 95% CI: 2.55 to 6.22) from 2013 to 2020 (Fig. 3, Table 2; Table S3, Supplemental Digital Content, https://links.lww.com/MD/P16). The Northeast depicted a decrease in the trajectory of the AAMR from 1999 to 2008 (APC: −6.69, 95% CI: −8.53 to −4.81) which stabilized thereafter till 2020 (APC: 0.80, 95% CI: −0.64 to 2.27) (Fig. 3, Table 2; Table S3, Supplemental Digital Content, https://links.lww.com/MD/P16).

**Figure 4.**
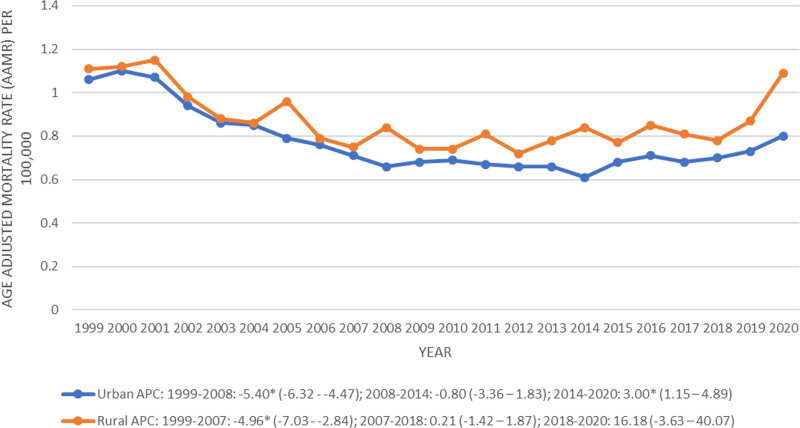
Peptic ulcer disease-related age-adjusted mortality rates stratified by urbanization per 100,000 among adults in the United States, 1999 to 2020 (APC = annual percentage change).

Rural regions exhibited a higher AAMR (0.86, 95% CI: 0.84 to 0.88) compared with the urban regions (0.76, 95% CI: 0.75 to 0.76) (Table 1). The rural regions showed a decrease in trends from 1999 to 2007 (APC: −4.96, 95% CI: −7.03 to −2.84), followed by a stable trajectory from 2007 to 2018 (APC: 0.21, 95% CI: −1.42 to 1.87) and from 2018 to 2020 (APC: 16.18, 95% CI: −3.63 to 40.07) (Fig. 4, Table 2; Table S4, Supplemental Digital Content, https://links.lww.com/MD/P16). From 1999 to 2008, the urban regions showed an appreciable decrease in the AAMR (APC: −5.40, 95% CI: −6.32 to −4.47), followed by a stable trajectory from 2008 to 2014 (APC: −0.80, 95% CI: −3.36 to 1.83) and finally a modest rise in trends from 2014 to 2020 (APC: 3.00, 95% CI: 1.15 to 4.89) (Fig. 4, Table 2; Table S4, Supplemental Digital Content, https://links.lww.com/MD/P16).

### 3.4. Trends by age group

Adults aged 85 years and older exhibited the highest CMR (7.63, 95% CI: 7.47 to 7.78) which was 5 times as much as the CMR depicted by the age group of 55 to 84 years (1.52, 95% CI: 1.50 to 1.54) and almost 48 times as much as the AAMR expressed by the age group of 25 to 54 years (0.16, 95% CI: 0.15 to 0.16) (Table 1).

**Figure 5.**
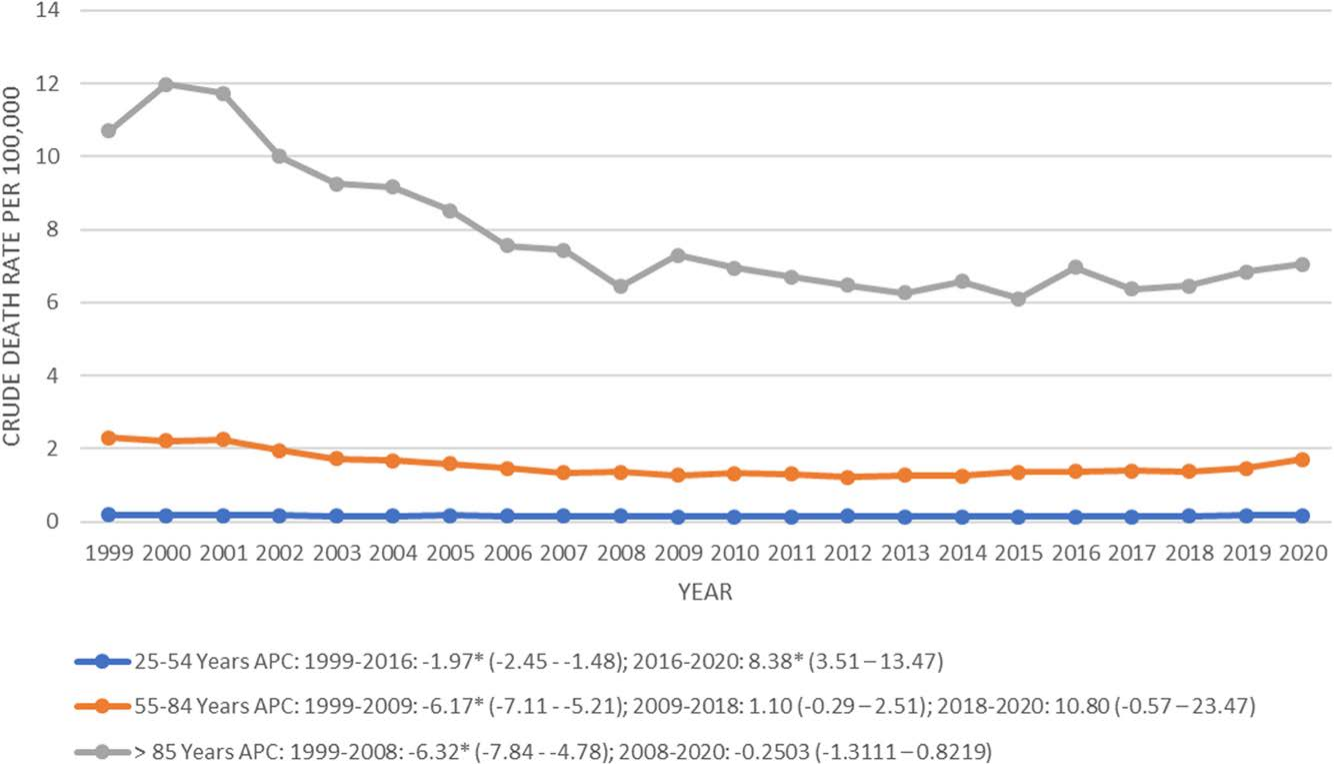
Peptic ulcer disease-related crude mortality rates stratified by age groups per 100,000 among adults in the United States, 1999 to 2020 (APC = annual percentage change).

A modest decrease in CMR was noted for all 3 age groups from 1999 to 2020, with the age group of 85 years and older expressing the most significant decrease in trends (AAPC: −2.90, 95% CI: −3.73 to −2.05), followed by the age group of 55 to 84 years (AAPC: −1.57, 95% CI: −2.72 to −0.41) and finally the age group of 25 to 54 years which depicted a stable trajectory throughout from 1999 to 2020 (AAPC: −0.07, 95% CI: −0.96 to 0.81) (Fig. 5, Table 2; Table S5, Supplemental Digital Content, https://links.lww.com/MD/P16). The age group of 85 years and older depicted a sharp decrease in CMR from 1999 to 2008 (APC: −6.32, 95% CI: −7.84 to −4.78), which remained stable onwards from 2008 to 2020 (APC: −0.25, 95% CI: −1.31 to 0.82) (Fig. 5, Table 2; Table S5, Supplemental Digital Content, https://links.lww.com/MD/P16). The age group of 55 to 84 years exhibited a decline in the AAMR from 1999 to 2009 (APC: −6.17, 95% CI: −7.11 to −5.21), and thereafter remained stable from 2009 to 2018 (APC: 1.10, 95% CI: −0.29 to 2.51) and from 2018 to 2020 (APC: 10.80, 95% CI: −0.57 to 23.47) (Fig. 5, Table 2; Table S5, Supplemental Digital Content, https://links.lww.com/MD/P16). The age group of 25 to 54 years showed a slight decrease in the trends of CMR from 1999 to 2016 (APC: −1.97, 95% CI: −2.45 to −1.48) before showing an abrupt rise in trends from 2016 to 2020 (APC: 8.38, 95% CI: 3.51 to 13.47) (Fig. 5, Table 2; Table S5, Supplemental Digital Content, https://links.lww.com/MD/P16).

### 3.5. Trends by place of death and state

**Figure 6.**
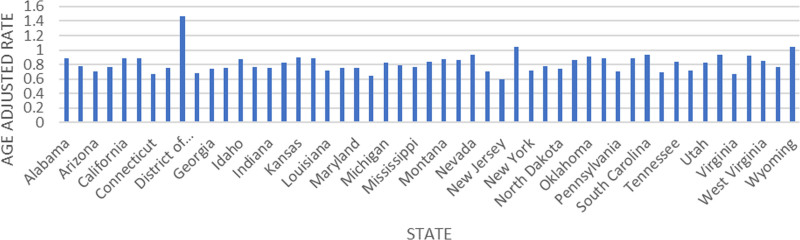
Peptic ulcer disease-related age-adjusted mortality rates stratified by state per 100,000 among adults in the United States, 1999 to 2020.

Most deaths (81.89%) occurred in medical facilities, followed by the decedent’s home (8.36%) and nursing home/long-term care (3.85%) (Table 1). There were significant differences in AAMRs across different states (Fig. 6). States with the highest AAMRs, such as District of Columbia (AAMR: 1.47; 95% CI: 1.21 to 1.73) and New Mexico (AAMR: 1.04; 95% CI: 0.92 to 1.15), exhibited rates approximately 2 times higher than those of states on the lower end of the spectrum, such as New Jersey (AAMR: 0.59; 95% CI: 0.55 to 0.63) and Massachusetts (AAMR: 0.65; 95% CI: 0.61 to 0.70) (Fig. 6).

## 4. Discussion

In this analysis of PUD-related mortality data in adults across 2 decades from the CDC WONDER database, we report several key findings. First, there was an initial period of stability in mortality rates from 1999 to 2001, following a significant decline from until 2012. A period of stability was observed from 2012 to 2018, followed by a significant rise from 2018 to 2020. This pattern was largely consistent among both older men and women. Second, males had higher mortality rates than females. Third, NH White adults had the highest PUD-related AAMR compared with other racial groups in this age strata. Fourth, residents of the District of Columbia, West and rural areas displayed the greatest mortality rates. Fifth, most of the deaths occurred in medical facilities. Lastly, older individuals had much higher mortality rates than younger ones.

An analysis of data from the National Inpatient Sample (NIS) database reveled a decline in PUD-related mortality in the United States from 2000 to 2011,^[12]^ which is fairly similar to our findings of a period of stability from 1999 to 2001 and a decline until 2012. Another study that used data from the NIS database revealed a decrease in the rate of decline in PUD-related mortality over the years from 2005 to 2014^[13]^ which supports our results. Over the past 2 decades, there have been many advancements in PUD diagnosis, treatment, and access to healthcare, as evidenced by the overall decline in mortality rates for both genders. Proton-pump inhibitors and H2 receptor antagonists are being used more widely, NSAIDs are used more responsibly, and H. pylori infections are declining, all of which have contributed to the decline in the age-standardized prevalence rate of PUD.^[1,14]^

However, our results report a peculiar increase from 2018 to 2020. A Mendelian randomization study revealed a causal relationship between body mass index (BMI) and the occurrence of gastric and peptic ulcers.^[15]^ This relationship could account for the increase seen in the latter years, as BMI has been on the rise in the US according to an analysis of long-term trends.^[16]^ Furthermore, this finding can also be linked to the COVID-19 pandemic. A study from Russia concluded the presence of coronavirus infection worsened preexisting gastrointestinal conditions including PUD.^[17]^ It is imperative to note that such changes, in the light of a pandemic, can exacerbate the preexisting burden on a healthcare system. It can devastate the provision of healthcare services, and require change in policies to deal with such an increase in morbidity and mortality.

We observed higher AAMRs in males in comparison to females. An analysis of the Global Burden of Disease study concluded higher prevalence, incidence, deaths, and disability-adjusted life years related to PUD in males in comparison to females over 29 years of age.^[18]^ Males have a higher prevalence of risk factors such as smoking in comparison to females,^[19]^ which has been associated with the development of PUD,^[15]^ and could explain the higher mortality rates in men compared to women.

The highest and lowest PUD-related AAMRs were found in NH Whites and Hispanics, respectively, which also show significant differences between racial and ethnic groups. Peptic and stomach ulcers are significantly positively correlated with smoking.^[20]^ An analysis of a survey found NH Whites to have the highest rate of cigarette consumption,^[21]^ suggesting a possible reason for the increased PUD-related AAMR among NH Whites. Efforts should be directed towards discouraging smoking through awareness campaigns, especially among groups at risk. This can be done by directing efforts towards patients admitted in or visiting the hospital for various conditions, as they may be more receptive to the information they receive about a risk factor, and are more likely to work towards quitting an addiction.

We observed higher CMRs for older adults as compared to the younger population. Recent studies of PUD patients with cirrhosis suggest that higher odds of in-hospital mortality are linked to increasing age and comorbidities.^[22,23]^ This difference can be explained by the range of various factors affecting mainly the geriatric population. The cause of PUD-related mortality can be attributed to the gastrointestinal bleeding, for which age is an independent risk factor.^[24]^ Gastrointestinal bleeding is linked to the use of NSAIDs and aspirin for the multiple underlying health conditions in the geriatric population.^[25]^ The declining renal function without dose adjustment pronounce the adverse effects of NSAIDs, leading to gastrointestinal hemorrhage.^[26]^ Other comorbid health conditions in older individuals—such as coronary artery disease and chronic renal sufficiency—are considered important risk factors for the development of PUD and its related complications.^[27]^ However, the mortality trends for all ages are decreasing, which can be attributed to the global awareness of the disease and the use of proton-pump inhibitors for H. pylori eradication.^[17]^ Strategies for awareness regarding NSAID use should also be formulated, and healthcare providers must be trained to educate patients to not use NSAIDs liberally or without prescription.

We observed a slight disparity between urban and rural population. This highlights that socioeconomic and demographic factors play a vital role in the prevalence of PUD and its outcomes.^[28]^ Urban areas have better access to endoscopy in contrast to rural settings, where lack of equipment and personnel are responsible for high morbidity and mortality.^[13]^ Guidelines which identify patients at risk and immediately refer patients at risk for a colonoscopy can help reduce mortality due to PUD. Furthermore, incentives for healthcare providers to train professionals present in rural settings can also help improve outcomes in these areas. It should be noted that individuals residing in rural areas could have a lower overall economic status in comparison to those residing in urban areas, which can further strain the ability to access healthcare and costly interventions such as endoscopy. Additionally, even if an individual may be financially able to undergo interventions, they might not be encouraged to do so if their social circle has never accessed advanced healthcare. However, both the subgroups revealed a surge in mortality rates after 2018, highlighting the impact of the COVID-19 pandemic. Fewer health visits, lack of follow-up care, and superimposed COVID-19 infection likely contributed to the overall mortality.^[29]^ Additionally, the West exhibited the highest AAMRS and the Northeast, the lowest. These findings align with another study conducted in 2017 through the NIS, which demonstrated the highest rates of peptic ulcer hemorrhage in the West, indicating a need to curb prevalence and mortality for different geographical regions.^[30]^

We also found the highest AAMRs to be exhibited by the District of Columbia, which has also previously been reported to exhibit the highest hepatitis B-related death rate from 2000 to 2019.^[31]^ Since hepatitis B virus X protein has been linked to the aggravation of gastric ulcers,^[32]^ this could explain the mortality rates seen in the District of Columbia.

We observed that the highest percentage of deaths occurred in medical facilities. Perceived stress is associated with the development of PUD^[33]^ and could explain the higher mortality rate observed in medical facilities due to stress associated with hospitalization. All patients, regardless of the initial risk assessment, should be routinely checked for signs of stress and presence of physical manifestations to identify the development of PUD or other gastrointestinal symptoms. This can ensure timely interventions to help reduce mortality.

To ensure that the suggestions made for reducing the morbidity and mortality burden due to PUD are successful, funding should be directed towards related programs such as awareness programs for smoking and training programs for endoscopy.

While we conducted a robust analysis using nationally representative data over 2 decades, our results should be interpreted considering some limitations. We used ICD-10 codes and death certificate data from the CDC WONDER database, which might lead to some missing cases. We could not adjust for comorbidities or socioeconomic factors, which likely affect the mortality rates. Lastly, the retrospective nature of our study limits the ability to prove causal relationships.

## 5. Conclusion

From 1999 to 2001 in the US, the mortality rates were stable, followed by a decline from 2001 to 2012, a period of stability thereafter before an increment from 2018 to 2020. The highest mortality rates were shown by males, NH Whites, residents of the West and rural areas, and older individuals. Future studies should try to delineate the causes of these trends and disparities. Timely implementation of control measures is necessary to reduce the burden of PUD-related deaths in the US.

## Author contributions

**Conceptualization:** Muhammad Babar, Muhammad Bilal Sardar, Arsalan Nadeem, Akash Gupta, Raheel Ahmed.

**Data curation:** Muhammad Babar, Mohsin Raza, Muhammad Asfandyar Nadir, Muhammad Bilal Sardar, Raheel Ahmed.

**Formal analysis:** Muhammad Babar, Mohsin Raza, Muhammad Asfandyar Nadir, Muhammad Bilal Sardar.

**Supervision:** Arsalan Nadeem, Akash Gupta, Raheel Ahmed.

**Writing – original draft:** Ariba Fida, Izzah Fayyaz, Armaghan Ur Rehman.

**Writing – review & editing:** Eeman Ahmad, Zain Ali Nadeem.

## Supplementary Material
