# Supplementary material for: Trends and disparities in peptic ulcer disease-related mortality in the United States from 1999 to 2020: A cross-sectional study
Source: Medicine (Baltimore). 2025 Oct 3;104(40):e42129. doi: 10.1097/MD.0000000000042129 (PMC12499665; doi:10.1097/MD.0000000000042129)
Supplement: Supplementary file 1 [file medi-104-e42129-s001.pdf]

SUPPLEMENTARY TABLE 1

Overall and Sex-stratified Peptic Ulcer Disease-related Age-adjusted Mortality Rates per 100,000 among Adults in the United States, 1999 to 2020

Overall	Year	Age Adjusted Rate	Age Adjusted Rate Lower 95% Confidence Interval	Age Adjusted Rate Upper 95% Confidence Interval	Age Adjusted Rate Standard Error
Overall	1999	1.06	1.02	1.11	1.02
Overall	2000	1.1	1.05	1.15	1.05
Overall	2001	1.09	1.04	1.13	1.04
Overall	2002	0.96	0.91	1	0.91
Overall	2003	0.87	0.83	0.91	0.83
Overall	2004	0.84	0.8	0.88	0.8
Overall	2005	0.81	0.77	0.85	0.77
Overall	2006	0.76	0.73	0.8	0.73
Overall	2007	0.72	0.68	0.76	0.68
Overall	2008	0.7	0.66	0.73	0.66
Overall	2009	0.69	0.66	0.73	0.66
Overall	2010	0.71	0.67	0.74	0.67
Overall	2011	0.68	0.65	0.72	0.65
Overall	2012	0.65	0.62	0.69	0.62
Overall	2013	0.67	0.63	0.7	0.63
Overall	2014	0.67	0.63	0.7	0.63
Overall	2015	0.69	0.66	0.73	0.66
Overall	2016	0.72	0.69	0.76	0.69
Overall	2017	0.7	0.66	0.73	0.66
Overall	2018	0.71	0.68	0.74	0.68
Overall	2019	0.76	0.73	0.8	0.73
Overall	2020	0.85	0.82	0.89	0.82
Female	1999	0.92	0.86	0.98	0.03
Female	2000	0.98	0.92	1.04	0.03
Female	2001	1.01	0.95	1.07	0.03
Female	2002	0.86	0.81	0.91	0.03
Female	2003	0.79	0.74	0.84	0.03
Female	2004	0.8	0.75	0.85	0.03
Female	2005	0.71	0.66	0.76	0.02
Female	2006	0.69	0.65	0.74	0.02
Female	2007	0.66	0.61	0.71	0.02
Female	2008	0.66	0.61	0.7	0.02
Female	2009	0.6	0.56	0.65	0.02
Female	2010	0.65	0.61	0.7	0.02
Female	2011	0.62	0.57	0.66	0.02

Female	2012	0.61	0.56	0.65	0.02
Female	2013	0.6	0.56	0.64	0.02
Female	2014	0.61	0.56	0.65	0.02
Female	2015	0.62	0.58	0.67	0.02
Female	2016	0.64	0.6	0.68	0.02
Female	2017	0.64	0.59	0.68	0.02
Female	2018	0.65	0.61	0.69	0.02
Female	2019	0.7	0.66	0.75	0.02
Female	2020	0.79	0.74	0.83	0.02
Male	1999	1.29	1.2	1.37	0.04
Male	2000	1.17	1.09	1.25	0.04
Male	2001	1.19	1.11	1.27	0.04
Male	2002	1.11	1.03	1.18	0.04
Male	2003	0.93	0.86	1	0.03
Male	2004	0.91	0.84	0.98	0.03
Male	2005	0.96	0.89	1.03	0.04
Male	2006	0.81	0.75	0.87	0.03
Male	2007	0.78	0.72	0.84	0.03
Male	2008	0.74	0.68	0.8	0.03
Male	2009	0.77	0.71	0.83	0.03
Male	2010	0.72	0.66	0.77	0.03
Male	2011	0.74	0.68	0.79	0.03
Male	2012	0.69	0.64	0.74	0.03
Male	2013	0.72	0.67	0.78	0.03
Male	2014	0.73	0.67	0.78	0.03
Male	2015	0.78	0.72	0.83	0.03
Male	2016	0.78	0.73	0.84	0.03
Male	2017	0.77	0.72	0.82	0.03
Male	2018	0.8	0.75	0.86	0.03
Male	2019	0.84	0.79	0.9	0.03
Male	2020	0.9	0.84	0.95	0.03

SUPPLEMENTARY TABLE 2

Peptic Ulcer Disease-related Age-adjusted Mortality Rates stratified by Race per 100,000 among Adults in the United States, 1999 to 2020

Race	Year	Age Adjusted Rate	Age Adjusted Rate Lower 95% Confidence Interval	Age Adjusted Rate Upper 95% Confidence Interval	Age Adjusted Rate Standard Error
NH Asian or Pacific Islander	1999	0.96	0.66	1.35	0.17
NH Asian or Pacific Islander	2000	0.68	0.44	0.99	0.13
NH Asian or Pacific Islander	2001	0.53	0.34	0.79	0.11
NH Asian or Pacific Islander	2002	0.74	0.51	1.04	0.13
NH Asian or Pacific Islander	2003	0.62	0.41	0.9	0.12
NH Asian or Pacific Islander	2004	0.64	0.43	0.91	0.12
NH Asian or Pacific Islander	2005	0.7	0.49	0.96	0.12
NH Asian or Pacific Islander	2006	0.52	0.35	0.74	0.1
NH Asian or Pacific Islander	2007	0.44	0.29	0.64	0.09
NH Asian or Pacific Islander	2008	0.55	0.38	0.77	0.09
NH Asian or Pacific Islander	2009	0.54	0.38	0.75	0.09
NH Asian or Pacific Islander	2010	0.48	0.33	0.67	0.08
NH Asian or Pacific Islander	2011	0.67	0.5	0.89	0.09
NH Asian or Pacific Islander	2012	0.41	0.29	0.58	0.07
NH Asian or Pacific Islander	2013	0.43	0.31	0.59	0.07
NH Asian or Pacific Islander	2014	0.38	0.26	0.52	0.06
NH Asian or Pacific Islander	2015	0.49	0.36	0.64	0.07
NH Asian or Pacific Islander	2016	0.37	0.26	0.5	0.06
NH Asian or Pacific Islander	2017	0.41	0.3	0.54	0.06
NH Asian or Pacific Islander	2018	0.48	0.36	0.63	0.07
NH Asian or Pacific Islander	2019	0.58	0.46	0.73	0.07

NH Asian or Pacific Islander	2020	0.53	0.42	0.67	0.06
NH Black or African American	1999	1.21	1.04	1.39	0.09
NH Black or African American	2000	1.13	0.97	1.3	0.09
NH Black or African American	2001	1.08	0.92	1.24	0.08
NH Black or African American	2002	0.8	0.67	0.94	0.07
NH Black or African American	2003	1	0.84	1.15	0.08
NH Black or African American	2004	0.9	0.76	1.04	0.07
NH Black or African American	2005	0.83	0.7	0.97	0.07
NH Black or African American	2006	0.82	0.69	0.96	0.07
NH Black or African American	2007	0.7	0.58	0.82	0.06
NH Black or African American	2008	0.65	0.54	0.77	0.06
NH Black or African American	2009	0.6	0.49	0.71	0.06
NH Black or African American	2010	0.6	0.49	0.71	0.05
NH Black or African American	2011	0.66	0.54	0.77	0.06
NH Black or African American	2012	0.67	0.56	0.78	0.06
NH Black or African American	2013	0.61	0.5	0.71	0.05
NH Black or African American	2014	0.67	0.56	0.77	0.06
NH Black or African American	2015	0.53	0.44	0.62	0.05
NH Black or African American	2016	0.75	0.63	0.86	0.06
NH Black or African American	2017	0.66	0.56	0.77	0.05
NH Black or African American	2018	0.72	0.62	0.83	0.06
NH Black or African American	2019	0.69	0.58	0.79	0.05
NH Black or African American	2020	0.78	0.67	0.88	0.06
NH White	1999	1.08	1.03	1.14	0.03
NH White	2000	1.08	1.02	1.13	0.03
NH White	2001	1.12	1.07	1.18	0.03

NH White	2002	0.98	0.93	1.03	0.03
NH White	2003	0.87	0.83	0.92	0.02
NH White	2004	0.86	0.81	0.9	0.02
NH White	2005	0.85	0.8	0.89	0.02
NH White	2006	0.76	0.72	0.8	0.02
NH White	2007	0.74	0.7	0.78	0.02
NH White	2008	0.72	0.68	0.76	0.02
NH White	2009	0.72	0.68	0.76	0.02
NH White	2010	0.74	0.7	0.78	0.02
NH White	2011	0.71	0.67	0.75	0.02
NH White	2012	0.69	0.65	0.73	0.02
NH White	2013	0.71	0.67	0.75	0.02
NH White	2014	0.71	0.67	0.75	0.02
NH White	2015	0.74	0.7	0.78	0.02
NH White	2016	0.76	0.72	0.8	0.02
NH White	2017	0.75	0.71	0.79	0.02
NH White	2018	0.76	0.72	0.81	0.02
NH White	2019	0.8	0.76	0.84	0.02
NH White	2020	0.92	0.87	0.96	0.02
Hispanic or Latino	1999	0.68	0.52	0.89	0.09
Hispanic or Latino	2000	0.72	0.55	0.92	0.09
Hispanic or Latino	2001	0.73	0.57	0.93	0.09
Hispanic or Latino	2002	0.76	0.59	0.95	0.09
Hispanic or Latino	2003	0.55	0.42	0.72	0.07
Hispanic or Latino	2004	0.64	0.5	0.81	0.08
Hispanic or Latino	2005	0.53	0.4	0.69	0.07
Hispanic or Latino	2006	0.46	0.35	0.61	0.06
Hispanic or Latino	2007	0.41	0.3	0.54	0.06
Hispanic or Latino	2008	0.47	0.37	0.6	0.06
Hispanic or Latino	2009	0.44	0.33	0.57	0.06
Hispanic or Latino	2010	0.42	0.32	0.54	0.05
Hispanic or Latino	2011	0.48	0.38	0.61	0.06
Hispanic or Latino	2012	0.47	0.37	0.59	0.05
Hispanic or Latino	2013	0.41	0.32	0.52	0.05
Hispanic or Latino	2014	0.37	0.29	0.47	0.04
Hispanic or Latino	2015	0.41	0.32	0.51	0.05
Hispanic or Latino	2016	0.41	0.33	0.51	0.04
Hispanic or Latino	2017	0.37	0.29	0.46	0.04
Hispanic or Latino	2018	0.47	0.38	0.56	0.05
Hispanic or Latino	2019	0.51	0.42	0.6	0.05
Hispanic or Latino	2020	0.54	0.44	0.63	0.05

Abbreviation: NH: Non-Hispanic

SUPPLEMENTARY TABLE 3

Peptic Ulcer Disease-related Age-adjusted Mortality Rates stratified by Census Region per 100,000 among Adults in the United States, 1999 to 2020

Census Region	Year	Age Adjusted Rate	Age Adjusted Rate Lower 95% Confidence Interval	Age Adjusted Rate Upper 95% Confidence Interval	Age Adjusted Rate Standard Error
Census Region 1: Northeast	1999	0.98	0.88	1.08	0.05
Census Region 1: Northeast	2000	1.11	1	1.22	0.06
Census Region 1: Northeast	2001	0.99	0.89	1.09	0.05
Census Region 1: Northeast	2002	0.94	0.84	1.03	0.05
Census Region 1: Northeast	2003	0.8	0.71	0.89	0.05
Census Region 1: Northeast	2004	0.8	0.72	0.89	0.04
Census Region 1: Northeast	2005	0.7	0.62	0.78	0.04
Census Region 1: Northeast	2006	0.57	0.5	0.65	0.04
Census Region 1: Northeast	2007	0.67	0.59	0.75	0.04
Census Region 1: Northeast	2008	0.6	0.52	0.67	0.04
Census Region 1: Northeast	2009	0.57	0.5	0.64	0.04
Census Region 1: Northeast	2010	0.66	0.58	0.74	0.04
Census Region 1: Northeast	2011	0.59	0.52	0.67	0.04
Census Region 1: Northeast	2012	0.56	0.49	0.63	0.04
Census Region 1: Northeast	2013	0.64	0.56	0.71	0.04
Census Region 1: Northeast	2014	0.59	0.52	0.67	0.04
Census Region 1: Northeast	2015	0.62	0.54	0.69	0.04
Census Region 1: Northeast	2016	0.58	0.51	0.66	0.04
Census Region 1: Northeast	2017	0.59	0.52	0.66	0.04
Census Region 1: Northeast	2018	0.63	0.55	0.7	0.04

Census Region 1: Northeast	2019	0.61	0.54	0.69	0.04
Census Region 1: Northeast	2020	0.72	0.65	0.8	0.04
Census Region 2: Midwest	1999	1.14	1.04	1.24	0.05
Census Region 2: Midwest	2000	1.11	1.01	1.21	0.05
Census Region 2: Midwest	2001	1.19	1.09	1.29	0.05
Census Region 2: Midwest	2002	1.03	0.93	1.12	0.05
Census Region 2: Midwest	2003	0.87	0.79	0.96	0.04
Census Region 2: Midwest	2004	0.79	0.7	0.87	0.04
Census Region 2: Midwest	2005	0.79	0.7	0.87	0.04
Census Region 2: Midwest	2006	0.81	0.73	0.89	0.04
Census Region 2: Midwest	2007	0.69	0.61	0.76	0.04
Census Region 2: Midwest	2008	0.71	0.64	0.79	0.04
Census Region 2: Midwest	2009	0.65	0.58	0.72	0.04
Census Region 2: Midwest	2010	0.74	0.66	0.81	0.04
Census Region 2: Midwest	2011	0.77	0.69	0.85	0.04
Census Region 2: Midwest	2012	0.72	0.64	0.8	0.04
Census Region 2: Midwest	2013	0.68	0.61	0.76	0.04
Census Region 2: Midwest	2014	0.61	0.54	0.67	0.03
Census Region 2: Midwest	2015	0.76	0.68	0.83	0.04
Census Region 2: Midwest	2016	0.76	0.68	0.83	0.04
Census Region 2: Midwest	2017	0.72	0.65	0.8	0.04
Census Region 2: Midwest	2018	0.74	0.66	0.81	0.04
Census Region 2: Midwest	2019	0.8	0.73	0.88	0.04
Census Region 2: Midwest	2020	0.94	0.86	1.02	0.04

Census Region 3: South	1999	0.98	0.91	1.06	0.04
Census Region 3: South	2000	1	0.93	1.08	0.04
Census Region 3: South	2001	1.03	0.95	1.11	0.04
Census Region 3: South	2002	0.85	0.77	0.92	0.04
Census Region 3: South	2003	0.87	0.8	0.94	0.04
Census Region 3: South	2004	0.82	0.75	0.88	0.03
Census Region 3: South	2005	0.82	0.75	0.89	0.03
Census Region 3: South	2006	0.74	0.68	0.8	0.03
Census Region 3: South	2007	0.75	0.69	0.82	0.03
Census Region 3: South	2008	0.74	0.67	0.8	0.03
Census Region 3: South	2009	0.68	0.62	0.73	0.03
Census Region 3: South	2010	0.64	0.58	0.69	0.03
Census Region 3: South	2011	0.63	0.57	0.69	0.03
Census Region 3: South	2012	0.65	0.59	0.7	0.03
Census Region 3: South	2013	0.6	0.55	0.65	0.03
Census Region 3: South	2014	0.65	0.59	0.7	0.03
Census Region 3: South	2015	0.63	0.58	0.69	0.03
Census Region 3: South	2016	0.73	0.68	0.79	0.03
Census Region 3: South	2017	0.71	0.66	0.77	0.03
Census Region 3: South	2018	0.72	0.66	0.77	0.03
Census Region 3: South	2019	0.74	0.69	0.8	0.03
Census Region 3: South	2020	0.83	0.77	0.89	0.03
Census Region 4: West	1999	1.34	1.22	1.46	0.06
Census Region 4: West	2000	1.2	1.08	1.31	0.06

Census Region 4: West	2001	1.16	1.05	1.27	0.06
Census Region 4: West	2002	1.16	1.05	1.27	0.06
Census Region 4: West	2003	0.88	0.78	0.97	0.05
Census Region 4: West	2004	1.03	0.93	1.13	0.05
Census Region 4: West	2005	0.97	0.88	1.07	0.05
Census Region 4: West	2006	0.87	0.78	0.96	0.05
Census Region 4: West	2007	0.73	0.65	0.81	0.04
Census Region 4: West	2008	0.71	0.63	0.79	0.04
Census Region 4: West	2009	0.82	0.73	0.9	0.04
Census Region 4: West	2010	0.79	0.71	0.87	0.04
Census Region 4: West	2011	0.78	0.7	0.86	0.04
Census Region 4: West	2012	0.73	0.66	0.81	0.04
Census Region 4: West	2013	0.78	0.7	0.86	0.04
Census Region 4: West	2014	0.79	0.72	0.87	0.04
Census Region 4: West	2015	0.75	0.68	0.83	0.04
Census Region 4: West	2016	0.75	0.67	0.82	0.04
Census Region 4: West	2017	0.76	0.68	0.83	0.04
Census Region 4: West	2018	0.83	0.75	0.9	0.04
Census Region 4: West	2019	0.84	0.77	0.92	0.04
Census Region 4: West	2020	0.86	0.78	0.93	0.04

SUPPLEMENTARY TABLE 4

Peptic Ulcer Disease-related Age-adjusted Mortality Rates stratified by Urbanization per 100,000 among Adults in the United States, 1999 to 2020

2013 Urbanization	Year	Age Adjusted Rate	Age Adjusted Rate Lower 95% Confidence Interval	Age Adjusted Rate Upper 95% Confidence Interval	Age Adjusted Rate Standard Error
Metropolitan	1.06	1.01	1.12	0.03	1.06
Metropolitan	1.1	1.04	1.15	0.03	1.1
Metropolitan	1.07	1.02	1.13	0.03	1.07
Metropolitan	0.94	0.9	0.99	0.02	0.94
Metropolitan	0.86	0.82	0.91	0.02	0.86
Metropolitan	0.85	0.81	0.9	0.02	0.85
Metropolitan	0.79	0.75	0.84	0.02	0.79
Metropolitan	0.76	0.72	0.81	0.02	0.76
Metropolitan	0.71	0.66	0.75	0.02	0.71
Metropolitan	0.66	0.62	0.7	0.02	0.66
Metropolitan	0.68	0.64	0.71	0.02	0.68
Metropolitan	0.69	0.65	0.73	0.02	0.69
Metropolitan	0.67	0.63	0.71	0.02	0.67
Metropolitan	0.66	0.62	0.7	0.02	0.66
Metropolitan	0.66	0.62	0.7	0.02	0.66
Metropolitan	0.61	0.58	0.65	0.02	0.61
Metropolitan	0.68	0.64	0.71	0.02	0.68
Metropolitan	0.71	0.67	0.74	0.02	0.71
Metropolitan	0.68	0.65	0.72	0.02	0.68
Metropolitan	0.7	0.66	0.73	0.02	0.7
Metropolitan	0.73	0.69	0.76	0.02	0.73
Metropolitan	0.8	0.76	0.84	0.02	0.8
Non-Metropolitan	1.11	0.99	1.22	0.06	1.11
Non-Metropolitan	1.12	1	1.23	0.06	1.12
Non-Metropolitan	1.15	1.03	1.26	0.06	1.15
Non-Metropolitan	0.98	0.87	1.08	0.05	0.98
Non-Metropolitan	0.88	0.78	0.98	0.05	0.88
Non-Metropolitan	0.86	0.76	0.95	0.05	0.86
Non-Metropolitan	0.96	0.86	1.07	0.05	0.96

Non-Metropolitan	0.79	0.7	0.89	0.05	0.79
Non-Metropolitan	0.75	0.66	0.84	0.05	0.75
Non-Metropolitan	0.84	0.74	0.93	0.05	0.84
Non-Metropolitan	0.74	0.65	0.82	0.04	0.74
Non-Metropolitan	0.74	0.65	0.83	0.05	0.74
Non-Metropolitan	0.81	0.72	0.9	0.05	0.81
Non-Metropolitan	0.72	0.64	0.81	0.04	0.72
Non-Metropolitan	0.78	0.69	0.87	0.05	0.78
Non-Metropolitan	0.84	0.75	0.93	0.05	0.84
Non-Metropolitan	0.77	0.68	0.85	0.04	0.77
Non-Metropolitan	0.85	0.76	0.94	0.05	0.85
Non-Metropolitan	0.81	0.72	0.9	0.05	0.81
Non-Metropolitan	0.78	0.7	0.87	0.04	0.78
Non-Metropolitan	0.87	0.78	0.96	0.05	0.87
Non-Metropolitan	1.09	0.98	1.19	0.05	1.09

SUPPLEMENTARY TABLE 5

Peptic Ulcer Disease-related Crude Mortality Rates stratified by Age Groups per 100,000 per 100,000 among Adults in the United States, 1999 to 2020

Age group	Year	Crude Rate	Crude Rate Lower 95% Confidence Interval	Crude Rate Upper 95% Confidence Interval	Crude Rate Standard Error
25-54 years	1999	0.19	0.16	0.21	0.01
25-54 years	2000	0.18	0.16	0.21	0.01
25-54 years	2001	0.18	0.16	0.21	0.01
25-54 years	2002	0.18	0.16	0.21	0.01
25-54 years	2003	0.16	0.14	0.19	0.01
25-54 years	2004	0.16	0.14	0.18	0.01
25-54 years	2005	0.17	0.14	0.19	0.01
25-54 years	2006	0.16	0.14	0.18	0.01
25-54 years	2007	0.16	0.14	0.18	0.01
25-54 years	2008	0.16	0.13	0.18	0.01
25-54 years	2009	0.14	0.12	0.17	0.01
25-54 years	2010	0.14	0.12	0.16	0.01
25-54 years	2011	0.13	0.11	0.15	0.01
25-54 years	2012	0.15	0.13	0.17	0.01
25-54 years	2013	0.14	0.12	0.16	0.01
25-54 years	2014	0.14	0.12	0.16	0.01
25-54 years	2015	0.14	0.12	0.17	0.01
25-54 years	2016	0.14	0.12	0.17	0.01
25-54 years	2017	0.13	0.11	0.15	0.01
25-54 years	2018	0.16	0.14	0.19	0.01
25-54 years	2019	0.17	0.15	0.19	0.01
25-54 years	2020	0.18	0.16	0.2	0.01
55-84 years	1999	2.31	2.18	2.44	0.07
55-84 years	2000	2.21	2.08	2.33	0.06
55-84 years	2001	2.25	2.13	2.37	0.06
55-84 years	2002	1.95	1.84	2.07	0.06
55-84 years	2003	1.72	1.61	1.82	0.05
55-84 years	2004	1.67	1.57	1.77	0.05
55-84 years	2005	1.59	1.5	1.69	0.05
55-84 years	2006	1.47	1.37	1.56	0.05
55-84 years	2007	1.34	1.25	1.42	0.05
55-84 years	2008	1.36	1.27	1.45	0.04
55-84 years	2009	1.27	1.19	1.36	0.04
55-84 years	2010	1.33	1.25	1.42	0.04
55-84 years	2011	1.31	1.23	1.39	0.04

55-84 years	2012	1.22	1.14	1.3	0.04
55-84 years	2013	1.27	1.19	1.35	0.04
55-84 years	2014	1.26	1.18	1.34	0.04
55-84 years	2015	1.36	1.28	1.44	0.04
55-84 years	2016	1.37	1.29	1.45	0.04
55-84 years	2017	1.39	1.31	1.47	0.04
55-84 years	2018	1.38	1.31	1.46	0.04
55-84 years	2019	1.47	1.4	1.55	0.04
55-84 years	2020	1.71	1.62	1.79	0.04
85+ years	1999	10.71	9.72	11.71	0.51
85+ years	2000	11.98	10.94	13.02	0.53
85+ years	2001	11.73	10.71	12.76	0.52
85+ years	2002	10	9.06	10.94	0.48
85+ years	2003	9.25	8.36	10.14	0.46
85+ years	2004	9.17	8.29	10.05	0.45
85+ years	2005	8.52	7.69	9.36	0.43
85+ years	2006	7.56	6.79	8.34	0.39
85+ years	2007	7.44	6.69	8.19	0.38
85+ years	2008	6.45	5.76	7.14	0.35
85+ years	2009	7.3	6.58	8.03	0.37
85+ years	2010	6.95	6.26	7.65	0.36
85+ years	2011	6.71	6.04	7.38	0.34
85+ years	2012	6.47	5.82	7.12	0.33
85+ years	2013	6.26	5.63	6.89	0.32
85+ years	2014	6.59	5.95	7.23	0.33
85+ years	2015	6.11	5.5	6.72	0.31
85+ years	2016	6.96	6.31	7.61	0.33
85+ years	2017	6.38	5.77	7	0.31
85+ years	2018	6.46	5.85	7.08	0.31
85+ years	2019	6.84	6.21	7.47	0.32
85+ years	2020	7.06	6.42	7.7	0.33
